# Learnings From a Pilot Study to Strengthen Primary Health Care Services: The Community-Clinic-Centered Health Service Model in Barishal District, Bangladesh

**DOI:** 10.9745/GHSP-D-20-00466

**Published:** 2021-03-15

**Authors:** Md. Eklas Uddin, Joby George, Shamim Jahan, Zubair Shams, Nazmul Haque, Henry B. Perry

**Affiliations:** a Save the Children, Dhaka, Bangladesh.; b Independent consultant, Hargeisa, Somalia.; c Johns Hopkins Bloomberg School of Public Health, Baltimore, MD, USA.

## Abstract

**Background:**

Community clinics (CCs) staffed by community health workers (CHWs) represent an effort of the Government of Bangladesh to strengthen the grassroots provision of primary health care services and to accelerate progress in achieving universal health coverage. The Improving Community Health Workers (ICHWs) Project of Save the Children piloted a CC-centered health service (CCHS) model that strengthened community and local government engagement, harmonized the work of different CHW cadres who were working in the same catchment area of each CC, and improved the accountability of CHWs and the CC to the local community.

**Methods:**

We describe the process for developing and implementing the CCHS model in 6 unions in Barishal District where the model was piloted and provide some early qualitative and quantitative findings pertaining to the model's effectiveness. Data were collected from CCs in the 6 pilot unions and 6 other unions that served as a control. Qualitative data were collected from the intervention area during the pre-pilot (October 2017–September 2018) and pilot phase (October 2018–September 2019). Document review, key informant interviews, and focus group discussions were also conducted. Maternal and child health service utilization data were extracted from the government health information system in both the intervention and control areas.

**Results:**

Community group meetings ensured engagement with local government authorities and supported resource mobilization. There was greater coordination of work among CHWs and increased motivation of CHWs to better serve their clients. The analysis showed that the increase in maternal health consultations was substantially greater in the intervention area than in the control area, as was the number of referrals for higher-level care.

**Conclusion:**

The CCHS model as applied in this pilot project is effective in engaging local key stakeholders, increasing CHW capacity, and improving client satisfaction. The model demonstrated that a community health system can be strengthened by a comprehensive approach that engages communities and local government officials and that harmonizes the work of CHWs.

## Abstract (precis)

The community-clinic-centered health service model piloted in Bangladesh strengthened community and local government engagement, harmonized the work of different community health worker cadres, and improved client satisfaction. The approach has the potential to strengthen the delivery of close-to-community primary health care services and accelerate progress toward achieving universal health coverage.

## Teaser

**Key Findings:**

- The process of creating a model to increase community health worker (CHW) harmonization and collaboration, increase community engagement, and improve quality of health services required multistakeholder platforms to develop policy, provide technical guidance, and implement interventions at the local level.
- Early qualitative findings of the pilot model found that there was a growing awareness of the need for the inclusion of the community as a key actor and a shared responsibility among CHWs, communities, and the local government in the process of community health system strengthening.
- Interventions implemented through the model increased resource mobilization for community clinic service delivery and the uptake of the primary health services at community level.

**Key Implications:**

- For policy makers, strengthening the delivery of community-centered primary health care services requires a comprehensive approach to engage both the community, CHWs, and local government.

## BACKGROUND

During the past 4 decades, Bangladesh has made a number of reforms in its health system and strengthened its extensive health service infrastructure in both the public and private sectors.^1^ As a result, Bangladesh has achieved impressive gains in population health, achieving the Millennium Development Goal 4 target of reducing under-5 child mortality by two-thirds between 1990 and 2015 and improvement in other key indicators such as maternal mortality, immunization coverage, and control of malaria, TB, and diarrhea diseases.^2^ Even so, all these indicators indicate that considerable challenges remain for reaching the health-related Sustainable Development Goal (SDGs).

Although the government's health system covers all citizens in theory, in practice, many sick people receive either no care or inadequate care.^1^ Private sector services are too expensive for many, and out-of-pocket expenditures for health care are high. The provision of services for the growing burden of noncommunicable diseases is only just beginning. Sixty-three percent of births still occur at home, often aided by unskilled birth attendants.^3^ Among noninstitutional deliveries, only 7% of newborns receive all 5 recommended essential newborn care practices.^3^ The coverages of antenatal care (ANC) and postnatal care services are still 82% and 52%, respectively. The quality of care in both public and private services is poor, with low levels of knowledge among service providers and poor application of skills in practice.^4^

In 1998, the Government of Bangladesh established community clinics (CCs) nationwide to improve access to essential primary health services to all, particularly for those in the most remote and hard-to-reach areas.^5^ By 2018, 13,779 CCs had been established with each serving 6,000–8,000 people in rural areas.

Despite the proliferation of CCs nationwide to improve access to essential primary health services, these services remained underutilized.^4^ The underutilization of CC services has been attributed to lack of awareness on the value of services; perceived poor quality of care; lack of coordination between CHWs of the 2 government directorates (health services and family planning), as well as between public sector and nongovernmental organization (NGO) CHWs who work at the CC and in its catchment areas; poor community engagement; lack of local government support for community health programs; and lack of accountability of service providers to the local community, among others.^6^^,^^7^

Despite the proliferation of community clinics nationwide to improve access to essential primary health services, these services remained underutilized.

Additional information about Bangladesh's community health system is provided in Supplement 1.

## IMPROVING COMMUNITY HEALTH WORKERS PROJECT

To increase the utilization of health services at CCs and to strengthen the community health system, the Improving Community Health Workers (ICHWs) Project (hereafter referred to as the Project) was launched in 2016. The Project is part of a 6-country global initiative funded by the U.S. Agency for International Development and the United Nations Children's Fund (UNICEF) that addresses recognized policy and implementation barriers to effective performance of CHWs. The overall goal of the global project is to help countries achieve full coverage, at the local level, of high-impact health and nutrition interventions. In Bangladesh, the Project was led by Save the Children International. The Project established a strategic partnership with UNICEF, BRAC (Bangladesh's largest NGO), and Partners in Health and Development, a local NGO that implemented the Project at the district level. The Project sought to create a model to encourage CHW collaboration, foster community engagement, and improve quality of health services.

Save the Children convened 3 multistakeholder platforms to operationalize the Project at the national and district levels.

1. It established a National Steering Committee, comprised of representatives from the government, development partners, and agencies to provide policy and strategic guidance, Project oversight, identification and endorsement of best practices, and incorporation of learnings into operational plans. Upon the committee's recommendation, the Ministry of Health and Family Welfare (MOHFW) approved a standardized definition for the role of CHWs along with a standardized job description to be applied to all CHW cadres. As a culmination of its policy-level work, the Project helped the MOHFW develop the Bangladesh National Strategy for Community Health Workers (2019–2030)^8^^–^^10^ (Box 1), providing clear guidance on CHWs' unique job functions and how they are to collaborate with and complement each other to enhance community health care services.
2. The Project established a National Stakeholder Forum to provide technical guidance and inputs into the CHW strategy and policy processes.
3. It established a District Coordination Committee composed of government personnel from local health and family welfare departments, NGO representatives, and other stakeholders including the local government to oversee and implement learning agenda activities at the local level.

BOX 1 **Ministry of Health and Family Welfare Community Engagement Strategy**

The current Bangladesh National Health Policy and strategies recognize active community engagement as a fundamental component of its health service system.^8^^–^^10^ Community health workers are at the interface between households, the community, and the health system and play a crucial role. The Ministry of Health and Family Welfare integrated the community engagement approach through the Operational Plan of Community-Based Health Care, which has, so far, formed about 13,800 community groups, each of which comprises members to support the management and operations of a community clinic. In addition, each community clinic has 3 community support groups. The community clinic policy promotes smooth, effective, and quality health services at the community level and defines mechanisms for engagement between each community clinic and the community it serves through the community groups and the community support groups.

In Barishal District, the Project established a District Learning Lab (DLL), covering a total of 187 CCs in 54 unions of 6 subdistricts, in conjunction with the District Coordinating Committee to strengthen Bangladesh's community health system. The DLL conducted a pilot test of a comprehensive service delivery and community organizing model—the CC-centered health service (CCHS) model. Through the DLL, stakeholders could influence the learning agenda, test and refine interventions, document findings, and collect evidence to guide national policy and program innovations.

There was a growing awareness of the need for the inclusion of the community as a key actor in the process of health systems strengthening.^11^ A pre-pilot analysis by Save the Children in 2018 on the CCHS model area recommended that the community groups (CGs) and community-support groups (CSGs) needed to be more functional and supportive of the CCs to foster stronger community engagement and accountability of service providers at each CC.

This article describes the Project's development of the CCHS model and its efforts toward health system strengthening through community and local government engagement.

## THE COMMUNITY-CLINIC-CENTERED HEALTH SERVICE MODEL

In supporting the DLL agenda, the Project designed a coordination and harmonization mechanism among CHWs, in conjunction with interventions for strengthening local government support, community engagement, and accountability. The Project piloted these interventions through the CCHS model from October 2018 to September 2019. The model aimed to provide a more coherent and organized approach to CHW programs leading to increased coverage, improved care-seeking, and increased referrals to higher levels of care. The CCHS model was tested in 25 CCs under 6 unions of 6 subdistricts of the DLL in Barishal District, Bangladesh.

The CCHS model aimed to provide a more coherent and organized approach to CHW programs to increase coverage of care, improve care-seeking behaviors, and increase referrals to higher levels of care.

Endorsed by the National Steering Committee and with the technical support from Save the Children, the CCHS model was implemented by a local partner NGO in collaboration with the District Coordination Committee.

**FIGURE 1:**
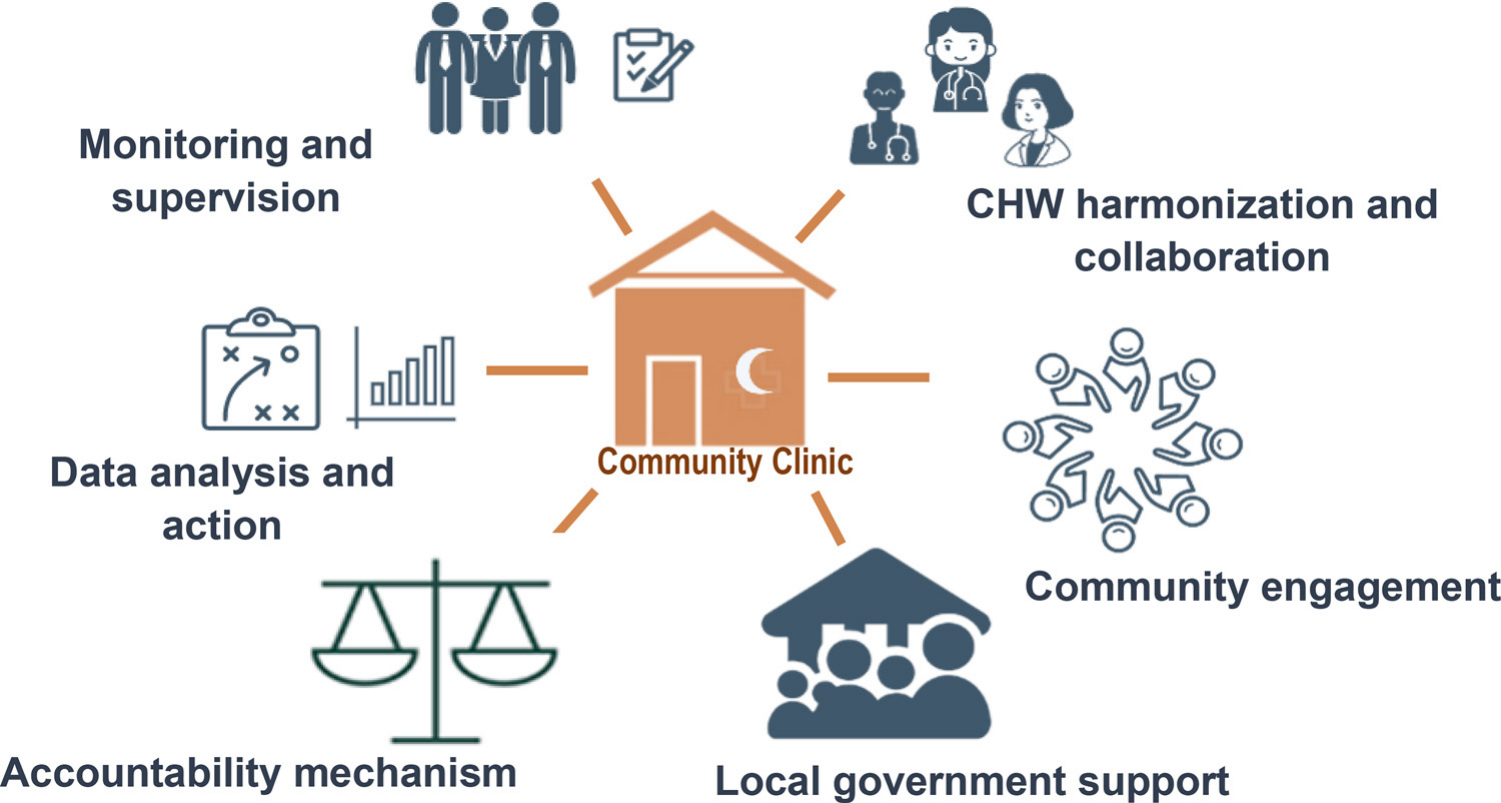
Components of the Community-Clinic-Centered Health Service Model Piloted in Bangladesh

Some of the key components for which the Project provided technical support are briefly described here (Figure 1).

### CHW Harmonization and Collaboration

The Project facilitated the development of harmonized job descriptions for the CHWs and tested them in the intervention area. The job descriptions contained the main general functions and tasks of all CHW cadres as well as the specific tasks in which each CHW cadre could coordinate with the other CHWs in the respective cadres, whether they were government or NGO CHWs. The harmonization process aimed to enable different cadres of CHWs to organize their work in a complementary fashion and facilitate alignment of their tasks. The Project organized a team training for CHWs during which 3 CHW cadres learned together. This helped them to support and coordinate with each other. The Project also facilitated microplanning meetings for each CC involving all CHWs, their supervisors, and community members to strengthen harmonization and cooperation.

### Community Engagement

One of the vital components of the CCHS model is community engagement. In the piloted CCs, the Project strengthened community engagement initiatives using different tools and techniques. According to the government community engagement protocol, the CG is pivotal in the management of the CC. The CG is a committee that includes 3 CHWs and has 17 members, most of whom are selected from the village. The head of the CG is an elected local government official.

In the catchment area of each CC, there are also 3 CSGs located in remote villages. Each CSG comprises 15–17 community members and 1–2 members who are also CG members. They support CG activities and promote CC message in their respective villages.

**FIGURE 2:**
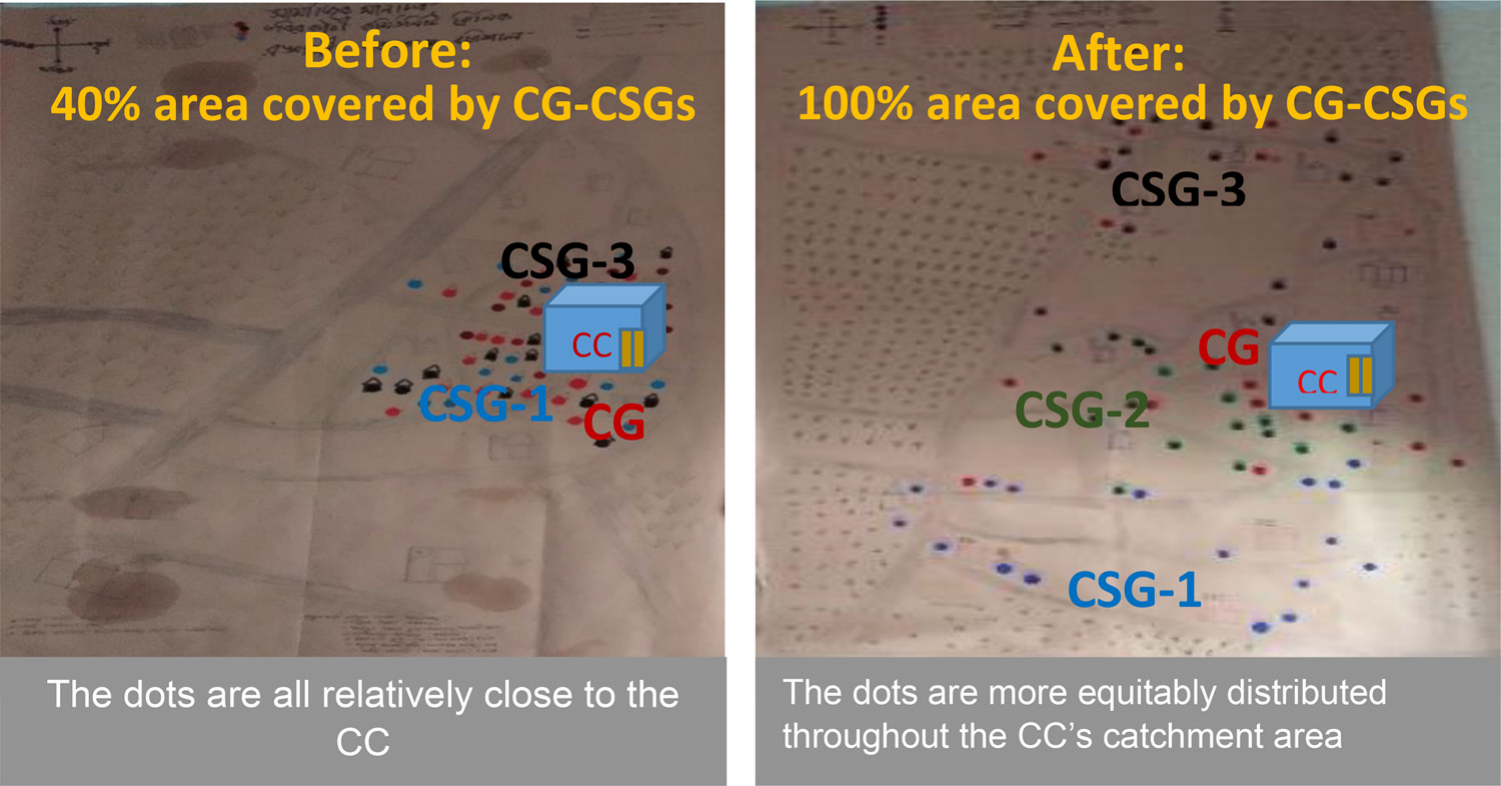
Social Map Showing Typical Distribution of Community Group and Community Support Group Members Before and After Reformation of Group Membership^a^ ^a^ Each dot refers to the residence location of a group member.

The members of the CGs and CSGs are supposed to be representative of the entire CC catchment area. Prepilot analysis in the CCHS model intervention area revealed that almost all the CG-CSG members were residing very close to the CC, and hardly any people were living in the outer portions of the CC catchment areas. In most cases, the number of members in each group was considerably less than called for, and most members were self-selected. As a consequence, the Project applied a social mapping tool to ensure representative membership of the entire community clinic catchment area and provided guidance to the members of the CGs and CSGs regarding their roles in CC management and operations. Figure 2 shows the change in residence location of CG and CSG members before and after reformation.

The Project applied a social mapping tool to ensure representative membership of the entire community clinic catchment area.

### Local Government Support

**FIGURE 3:**
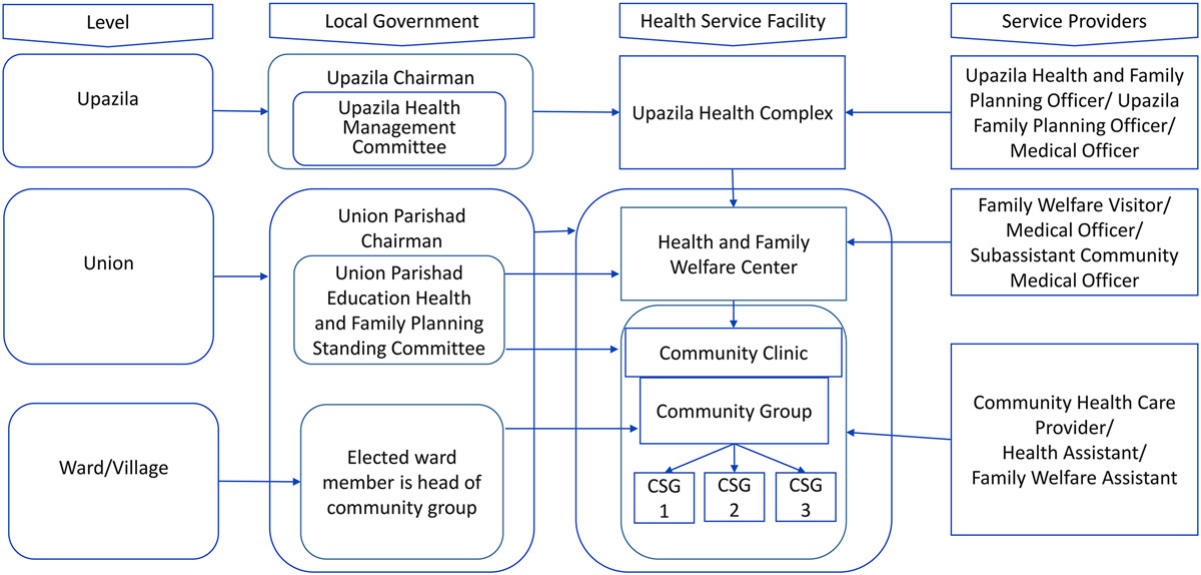
Organizational Hierarchy and Relationship Between Local Government and Rural Community Health Service Delivery System, Bangladesh Abbreviation: CSG, community support group.

The union parishad (UP) is the lowest government administrative unit in rural Bangladesh. Each UP is made up of 9 wards, with usually 1 village designated as a ward. Each UP is supposed to form a health, education, and family planning standing committee to oversee community health functions. Figure 3 shows the organizational hierarchy and relationships between local government (UP) and the community health service delivery system. In addition, each UP is expected to allocate 10%–15% of its annual budget for health issues. The elected UP member of the ward where a CC is situated is expected to serve as the chairperson of the CG. Evidence suggests that active participation of UP members as chairs of CGs facilitates good performance of CGs, and a well-functioning CG makes CC health workers more accountable.^5^ So, the Project organized orientations for UPs and facilitated knowledge sharing, best practices, and advocacy that aimed to ensure the UP's support for community health programs.

### Accountability Mechanisms

The community scorecard is a powerful tool to monitor services, empower citizens, and improve the accountability of service providers. The scoring exercises provide citizens the opportunity to analyze health services based on their personal perceptions.^12^ The Project introduced the community scorecard tool in the intervention area to obtain citizen perceptions about CC services, hold the service providers accountable, and ultimately improve quality of CC services. The Project implemented the community scorecard in 13 CCs. The Project's technical team customized and facilitated the community scorecard process.

### Monitoring, Supervision, and Data Analysis

The Project also facilitated the government's existing procedures for monitoring and supervision, data analysis, and implementation of findings.

Supplement 2 provides further details about the implementation process of and learning from CCHS model components.

## METHODS

### Study Sites

The CCHS model was implemented in the intervention area in Barishal District, located in southern Bangladesh. The district was selected because of its high disease burden and low income. According to a Bangladesh Demographic and Health Survey (2014), in Barishal District, 69% of deliveries occur at home; and the crude birth rate is 22.2 per 1,000 populations per year.^13^ Save the Children has had a longstanding engagement in this region with health, education, and humanitarian support programs. In addition, the MOHFW authorities maintain good coordination with NGOs and cooperate with any new program interventions that enhance the community health system.

The CCHS Model and its assessment were carried out in 6 UPs (1 UP from each upazila) in the Project area. Another 6 UPs outside of the Project area were selected from adjacent upazilas as a control area (Table 1). The control areas were located geographically adjacent to the intervention areas and shared similar socioeconomic characteristics.

**TABLE 1.** Study Intervention and Control Areas of the Community-Clinic-Centered Health Service Model Pilot Intervention, Bangladesh

Intervention Area District	Upazila	Union Parishad	District	Control Area Upazila	Union Parishad
Barishal	Babuganj	Rahamatpur	Barishal	Agailjhara	Goila
	Bakerganj	Rangashree			Rajihar
	Gaurnadi	Mahilara		Muladi	Charkalekhan
	Banaripara	Soliabagpur			Kazirchar
	Wazirpur	Bamrail	Patuakhali	Sadar	Laukathi
	Sadar	Raypasha korapur			Borobighai

The UPs in the intervention area were selected by the District Coordination Committee and the Upazila Health and Family Planning Officer on the basis of the following considerations: previous service performance, availability of human resources (CHWs present as well as their supervisors), presence of NGO community health workers, availability of infrastructure and communication (UPs in areas where communication is easy as well as UPs in hard-to-reach areas), and Government of Bangladesh consensus.

### Data Collection Methods

The study collected both qualitative and quantitative data from both the intervention and control areas.

#### Primary Data Collection

Key informant interviews and focus group discussions were conducted using a structured questionnaire. Interviews were done in each UP in both the intervention and control areas. The respondents were selected purposely, and all the interviews were conducted in person (Table 2).

**TABLE 2.** Qualitative Data Collection of the Community-Clinic-Centered Health Service Model Pilot Intervention, Bangladesh

Type of Respondent	Respondents	
Community health worker supervisors	Assistant Health Inspector	12 key informant interviews
	Expanded Program on Immunization	12 key informant interviews
	Supervisor: nongovernmental organization	12 key informant interviews
Health service managers	Upazila Health and Family Planning Officer	9 key informant interviews
	Upazila Family Planning Officer	9 key informant interviews
Local government representative	Union parishad chairman/ Union Parishad Education, Health & Family Planning Standing Committee member	12 key informant interviews
Total		66 persons interviewed
Community representative	Community group and community support group member	12 focus group discussions (96 total focus group discussion participants)

#### Secondary Data Collection

We conducted an extensive record review and data extraction from the government health information system since the activities of CCs are included in this. Project-level reports including baseline and endline assessment and policy briefs were also reviewed. For our purposes here, we use the term prepilot phase as the period of collection of baseline data (October 2017–September 2018) while the pilot phase refers to the period of Project intervention, October 2018–September 2019.

### Data Analysis

Themes observed in the qualitative data were identified manually and then converted into codes and subcodes by aligning them with key components of the CCHS model. For quantitative data, we calculated descriptive statistics and 2-sample t-tests with equal variances using Microsoft Excel.

### Ethical Considerations

The study proposal was reviewed by the Save the Children Ethics Review Committee and determined to be exempt from further human subjects review. During all interviews, the interviewers obtained verbal consent and assured respondents that there would be no adverse physical or psychological effects from their participation. The interviewers also informed the respondents that they could leave the interview anytime and that they did not necessarily have to answer all questions. The study team followed the Save the Children policy about receiving prior permission for taking and using visual still or moving images. All the responses were de-identified before analysis.

## RESULTS

### Qualitative Assessment Findings

From the interviews, it was found that the key stakeholders became more involved in the CG/CSG meetings (Box 2). Details are as follows.

BOX 2 **Summary of Comments From Key Informant Interviews and Focus Group Discussions Regarding the Effectiveness of the Project Intervention**

- Improvement in quality of the daily work of community health workers
- More coordination
- Greater sense of sharing and cooperation among providers
- Reduced data duplication
- Easier to identify and correct mistakes
- Increased accountability to each other and to the community
- Improved coordination between government and nongovernmental organization health workers
- Gaps in service coverage reduced
- Increased information sharing
- Health services improved
- Community health workers better able to reach their targets
- Greater transparency of work
- Greater efficiency of work, providing time to devote to improving the quality of services

Respondents mentioned that joint planning meetings held between the government and NGO workers made it possible to share reports, reduce under- and overreporting of services, and reduce duplication in the reporting of services. CHW supervisors mentioned that CHWs conducted and attended joint planning and report sharing meetings regularly as part of the community microplanning meetings that aimed to ensure that the entire population in the UP was reached. The microplanning meetings provided a useful mechanism to bring the health and family planning CHWs together with the community to accurately capture community information and ensure that consistent data were being reported.

Microplanning meetings for each CC between the CHWs, supervisors, and community provided a useful mechanism to bring all CHWs together and ensure accurate community data were captured.

The Project found that some CGs and CSGs in pilot areas were not formed according to MOHFW guidelines—a key reason why those committees were not functioning well. In the intervention area, all 25 CGs and 75 CSGs were formed following the proper guidelines (i.e., representing the whole population). The Project provided technical assistance to CCs in helping to update and reform 25 CGs and 75 CSGs by selecting members from all catchment areas of 25 CCs within the intervention area using a community mapping tool. CG meetings were attended by most of the CHWs, and all CSG meetings were attended by one of the CHWs.

CHWs mentioned that their activities were overseen by the local government representatives. The local government played an important role in promoting CC and CHW services in the community and provided additional support needed for the improvement of the CC's infrastructure.

The CHW supervisors and the health and family planning managers monitored all the CHWs' activities and provided them with feedback. They also helped the CHWs provide health services for mothers and children. During supervision, they checked the record books and helped in developing strategies for re-engaging clients who were in need of additional services. The health and family planning managers also conducted a joint supervisory visit to each CC every month and complete a supervisory checklist. In the next visit, the identified issues were followed up with the respective staff, leading to a significant improvement in the quality of services.

The community scorecards contributed to improved quality, efficiency, and accountability of services and provided useful information on medicine shortages, service provider behaviors, and other client complaints. There was a general agreement among all the interviewees that the community scorecards had been effective in enabling clients to ask for and receive more effective and respectful care. The community scorecards also strengthened the accountability of CHWs to the community and motivated CHWs to achieve a higher level of excellence in their work.

### Quantitative Assessment Findings

**FIGURE 4:**
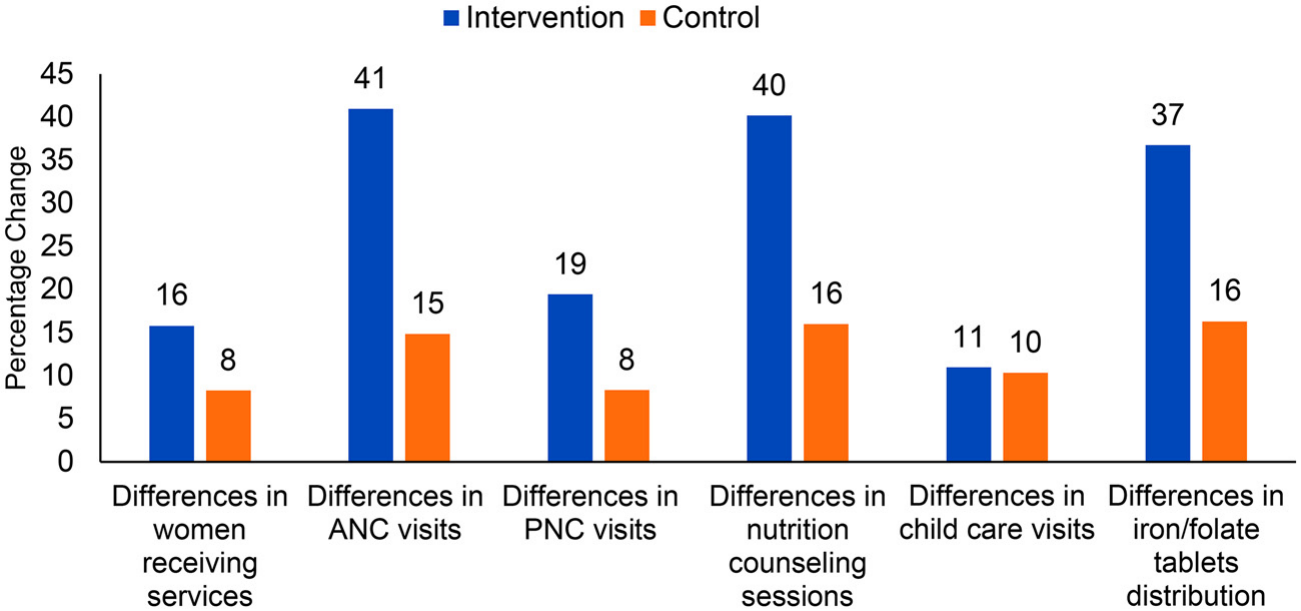
Difference in Maternal and Child Health Community Clinic Services Utilization Between the Pre-pilot Period (October 2017–September 2018) and the Community-Clinic-Centered Health Service Model Pilot Period (October 2018–September 2019), Barishal District, Bangladesh Abbreviations: ANC, antenatal care; PNC, postnatal care.

Between the prepilot and pilot phases, the number of women in UPs in the intervention areas using services (Figure 4) increased more compared to the number of women in UPs in the control areas. In the intervention areas, visits for ANC, postnatal care, and nutrition counseling, as well as iron/folate tablets distributed, increased more compared to the control areas. However, there was no difference between the intervention and control areas in child health services provided. Supplement 3 provides more detailed information on services provided and statistical significance for all of the quantitative data shown. Three of the five difference-in-differences (for antenatal care, nutritional counseling, and iron/folate distribution) were statistically significant at the 0.01 level.

**FIGURE 5:**
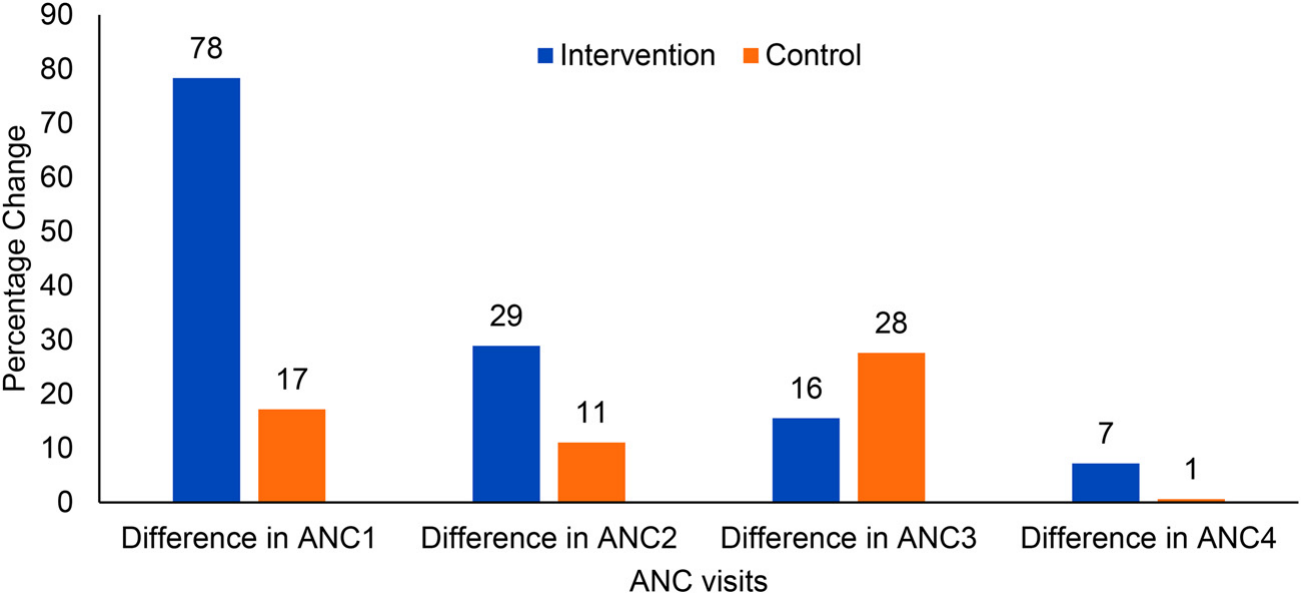
Difference in Antenatal Care Visits at Community Clinics (%) Between the Pre-pilot Period (October 2017–September 2018) and the Community-Clinic-Centered Health Service Model Pilot Period (October 2018–September 2019), Barishal District, Bangladesh

Between the prepilot and pilot phases, coverage of ANC visits increased in the UPs in intervention areas (Figure 5) more compared to those in control areas, with the greatest increase in ANC visits being for the first ANC visit (ANC1). There was improvement in utilization for ANC2 and ANC3 visits as well, but the difference-in-differences progressively declined from ANC1 to ANC4. There was minimal change in the coverage of 4 ANC visits in the intervention and control areas.

**FIGURE 6:**
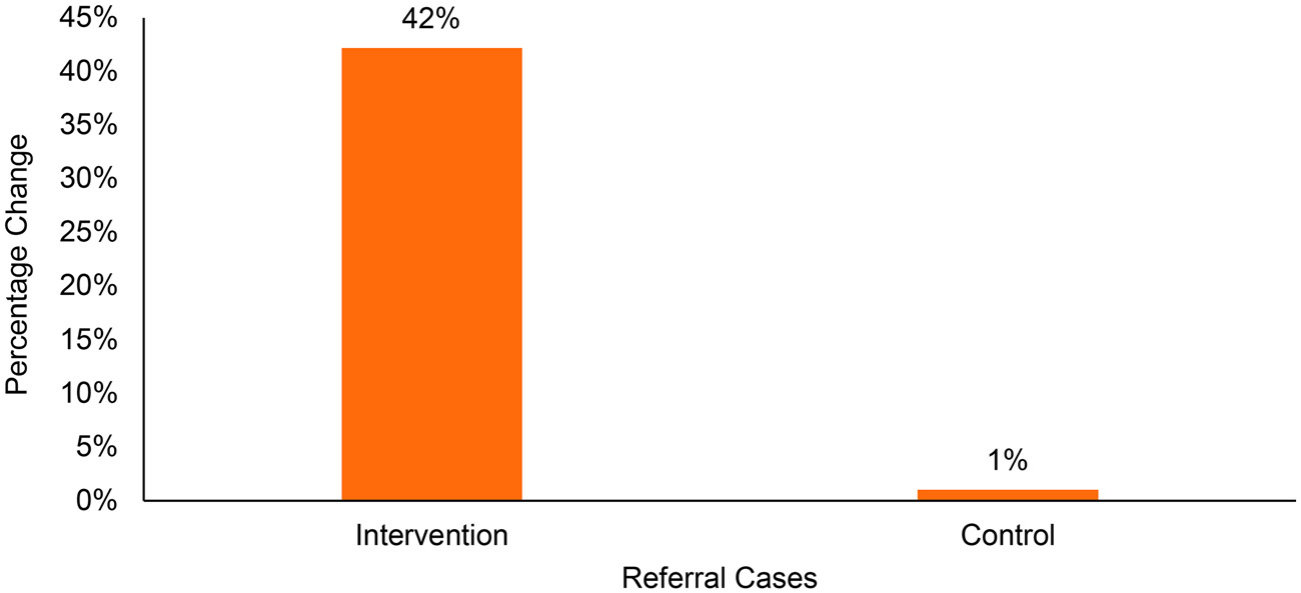
Difference in Referral Cases From Community Clinics Between the Pre-pilot Period (October 2017–September 2018) and the Community-Clinic-Centered Health Service Model Pilot Period (October 2018–September 2019), Barishal District, Bangladesh

Between the prepilot and pilot phases, referrals of mothers and children for higher levels of care increased 42% in UPs in the intervention area compared to increasing 1% in the control area (Figure 6).

## DISCUSSION

We have described a pilot intervention model for improving the functioning of the local-level community health system in 6 UPs of the Barishal District, Bangladesh. Through a process of strengthening the formation and functioning of CGs and CSGs and the installation of microplanning meetings that enabled CHWs to better coordinate their work, increases in the utilization of maternal health services were observed. Increases in the first ANC visit were particularly pronounced. In addition, there were marked increases in the number of women referred from CCs in the intervention area compared to the control area. The increase in referrals reflects CHWs' improved capacity to make decisions about women needing higher levels of care. However, the same cannot be said of utilization of child health services. It may be due to facility readiness gaps for providing childcare services like consistent availability of expanded program of immunization, relevant medicines for fever or vomiting. Also, CCs might not be as child-friendly because of lack of space or poster/cartoon for child communication.

The Project facilitated monthly community microplanning meetings in the intervention area and introduced key stakeholders to how these meetings could increase the efficiency and effectiveness of their work. The participation of CHWs, their supervisors, UP representatives, and community members helped to make these meetings effective. Gradually, CHWs became more comfortable sharing data and addressing common challenges during these meetings. Similarly, research from Sierra Leone by O'Connor et al. showed that CHWs' sharing of their data with local health system and community government representatives can lead to improvements in health system functioning.^14^

This study suggests that the interventions employed for strengthening the role of the CGs and CSGs and for improving the harmonization and coordination of CHWs in the catchment areas of the CCs were effective in improving the utilization of services for pregnant women. Similar results were also observed in other countries. Namazzi et al. showed that CHWs in Ghana generated an increased utilization of maternal and neonatal health services through their active participation in the community and their capacity to refer high-risk cases to nearby facilities.^15^ Similar to the CGs and CSGs platform, Awasthi et al. suggested that community engagement and social awareness could help in promoting the utilization of maternal health services.^16^

The current study supports the conclusion that the CCHS model enables both UPs as well as CGs and CSGs to be more involved in CC oversight and accountability monitoring. The model also leads to improved coordination and effectiveness of the CHWs working in the CC catchment area. These, in turn, contribute to an increase in utilization of services at the CCs. A comprehensive approach was required to achieve these results. However, the functionality of local government committees varies.

The study demonstrates that engaging the community can help to resolve longstanding issues in community health programs. The social mapping process helped make CGs and CSGs more representative and more effective. The community scorecard tool ensured tripartite coordination among CHWs, the community, and the local government, and enhanced accountability of services to the community. In the Democratic Republic of the Congo, community scorecards bridged the divisions between frontline health care providers and community members by providing opportunities for exchange and collaboration at the community level.^17^ Community scorecards can increase the availability of information about maternal and neonatal health services.^18^ Effective facilitation of the community scorecard process requires skilled application, but the use of community scorecards needs to be expanded.

The community scorecard tool ensured tripartite coordination among CHWs, the community, and the local government and enhanced accountability of services to the community.

The Bangladesh National Strategy for Community Health Workers (2019–2030),^10^ developed in part on the basis of the experiences gained by implementation of the Project, needs to be implemented and scaled up. Ongoing monitoring and evaluation are needed. Our study suggests that local stakeholders and CHWs will need to work together for this strategy to be successful.

### Limitations

Our study has some limitations. The quantitative outcomes assessed here are limited to utilization of services at the CC. We did not carry out any assessments regarding whether the Project had any impact on quality of care or on population coverage of services. In addition, the qualitative data assessment included data only during the first 6 months of the intervention (pilot) phase (October 2018–March 2019) because the COVID-19 pandemic had affected the intervention area at that time, prohibiting the collection of additional qualitative data.

## CONCLUSION

In Bangladesh, there is a need to address the fragmentation of different CHW programs and to harmonize their work at the micro level. There is also a great need to engage communities in the oversight of the community health system. Innovative community-based strategies such as the ones described here and implemented on a pilot basis can be useful for guiding the rollout of interventions and health improvements at scale. Government and civil society organizations are well placed to reduce fragmentation and duplication of CHW services and to establish a more effectively functioning local health team through community partnerships.

The next decade will be critical to cement Bangladesh's community-based health care system as the foundation of its primary health care system and achieve the SDGs and UHC by 2030. Building on previous learnings and achievements, the community health system now has the opportunity to become stronger. It is now widely recognized that CHWs have been major providers of the essential service package and have the potential for increasing their uptake even further. Therefore, to maximize their reach and effectiveness, the Government of Bangladesh will need to continue to invest in CHW support using methods such as those that have been piloted in this Project.

## Supplementary Material

20-00466-Uddin-Supplement3.pdf

20-00466-Uddin-Supplement2.pdf

20-00466-Uddin-Supplement1.pdf

## Notes

**Peer Reviewed**
