# Supplementary material for: Learnings From a Pilot Study to Strengthen Primary Health Care Services: The Community-Clinic-Centered Health Service Model in Barishal District, Bangladesh
Source: Glob Health Sci Pract. 2021 Mar 15;9(Suppl 1):S179–89. doi: 10.9745/GHSP-D-20-00466 (PMC7971368; doi:10.9745/GHSP-D-20-00466)
Supplement: 20-00466-Uddin-Supplement3.pdf [file 20-00466-Uddin-Supplement3.pdf]

Supplement to: Uddin ME, George J, Jahan S, Shams Z, Haque N, Perry HB. Learnings from a pilot study to strengthen primary health care services: the community-clinic-centered health service model in Barishal District, Bangladesh. *Glob Health Sci Pract*. 2021;9(Suppl 1). https://doi.org/10.9745/GHSP-D-20-00466

Supplement 3.

Difference in difference analysis on selected key health indicators in between pre-pilot phase and pilot phase (by comparing means for each indicator in the Intervention and Comparison Areas using 2 sample t-test (Supplement 3. Table 1)

Supplement 3. Table 1. Numbers of specific types of services provided in the Community Clinics in the Intervention Area during the Pre-Pilot Phase (October 2017-September 2018) and the Pilot Phase (October 2018-September 2019)

**Services**	**Pre-pilot phase**	**Pilot phase**	**Difference**	**Mean, standard deviation**	**P value***
**Number of women receiving care**					0.06
Intervention Area					
Mahilara	1719	2002	283		
Rangasree	2727	2851	124		
Soliabagpur	1469	2059	590		
Bamrail	1398	1758	360		
Raypasha	1491	1537	46		
Rahmatpur	1390	1598	208		
Mean and standard deviation of the differences				268.5, 192.87	
Control Area					
Gaila	809	965	156		
Rajihar	1528	1293	-235		
Charkalehan	953	1716	763		
Kazichor	1031	1101	70		
Laukathi	2514	2508	-6		
Borobighai	1398	1332	-66		
Mean and standard deviation of the differences				113.67,344.53	
**Antenatal care**					0.004
Intervention Area					
Mahilara	314	422	108		
Rangasree	871	916	45		
Soliabagpur	364	826	462		
Bamrail	591	945	354		
Raypasha	386	435	49		

Supplement to: Uddin ME, George J, Jahan S, Shams Z, Haque N, Perry HB. Learnings from a pilot study to strengthen primary health care services: the community-clinic-centered health service model in Barishal District, Bangladesh. *Glob Health Sci Pract*. 2021;9(Suppl 1). https://doi.org/10.9745/GHSP-D-20-00466

**Services**	**Pre-pilot phase**	**Pilot phase**	**Difference**	**Mean, standard deviation**	**P value***
Rahmatpur	391	568	177		
Mean and standard deviation of the differences				199.0,171.2	
Control Area					
Gaila	238	280	42		
Rajihar	470	399	-71		
Charkalehan	295	517	222		
Kazichor	236	410	174		
Laukathi	495	493	-2		
Borobighai	286	221	-65		
Mean and standard deviation of the differences				50.0, 123.0	
**Postnatal care**					0.23
Intervention Area					
Mahilara	167	182	15		
Rangasree	238	258	20		
Soliabagpur	95	131	36		
Bamrail	172	195	23		
Raypasha	118	135	17		
Rahmatpur	65	120	55		
Mean and standard deviation of the differences				27.7, 15.3	
Control Area					
Gaila	85	84	-1		
Rajihar	107	86	-21		
Charkalehan	124	227	103		
Kazichor	69	105	36		
Laukathi	266	241	-25		
Borobighai	141	115	-26		
Mean and standard deviation of the differences				11.0, 50.8	
**Nutritional counselling**					0.01
Intervention Area					
Mahilara	321	344	23		
Rangasree	670	682	12		
Soliabagpur	361	821	460		
Bamrail	584	940	356		
Raypasha	373	438	65		
Rahmatpur	378	542	164		

Supplement to: Uddin ME, George J, Jahan S, Shams Z, Haque N, Perry HB. Learnings from a pilot study to strengthen primary health care services: the community-clinic-centered health service model in Barishal District, Bangladesh. *Glob Health Sci Pract*. 2021;9(Suppl 1). https://doi.org/10.9745/GHSP-D-20-00466

Services	Pre-pilot phase	Pilot phase	Difference	Mean, standard deviation	P value*
Mean and standard deviation of the differences				180.0, 187.47	
Control Area					
Gaila	232	242	10		
Rajihar	459	395	-64		
Charkalehan	245	489	244		
Kazichor	222	313	91		
Laukathi	465	459	-6		
Borobighai	184	198	14		
Mean and standard deviation of the differences				44.2, 108.0	
**Iron/folic acid tablet distribution**					0.01
Intervention Area					
Mahilara	475	509	34		
Rangasree	847	830	-17		
Soliabagpur	450	945	495		
Bamrail	742	1124	382		
Raypasha	473	591	118		
Rahmatpur	435	680	245		
Mean and standard deviation of the differences				209.5, 201.5	
Control Area					
Gaila	317	344	27		
Rajihar	563	483	-80		
Charkalehan	359	643	284		
Kazichor	284	411	127		
Laukathi	628	685	57		
Borobighai	361	356	-5		
Mean and standard deviation of the differences				68.3, 125.8	
**Number of children younger than 5 years of age receiving services**					0.39
Intervention					
Mahilara	2782	3300	518		
Rangasree	3438	3874	436		
Soliabagpur	4762	4276	-486		
Bamrail	2918	4176	1258		
Raypasha	2670	2976	306		

Supplement to: Uddin ME, George J, Jahan S, Shams Z, Haque N, Perry HB. Learnings from a pilot study to strengthen primary health care services: the community-clinic-centered health service model in Barishal District, Bangladesh. *Glob Health Sci Pract*. 2021;9(Suppl 1). https://doi.org/10.9745/GHSP-D-20-00466

**Services**	**Pre-pilot phase**	**Pilot phase**	**Difference**	**Mean, standard deviation**	**P value***
Rahmatpur	2200	2230	30		
Mean and standard deviation of the differences				343.7, 576.7	
Control					
Gaila	2288	2540	252		
Rajihar	2830	2364	-466		
Charkalehan	3322	3904	582		
Kazichor	2922	4136	1214		
Laukathi	2822	2530	-292		
Borobighai	920	1194	274		
Mean and standard deviation of the differences				258, 679.4	
**Number of referrals of women and children**					0.07
Intervention	53	203	150		
Mahilara	79	89	10		
Rangasree	46	59	13		
Soliabagpur	166	174	8		
Bamrail	17	28	11		
Raypasha	2	6	4		
Rahmatpur					
Mean and standard deviation of the differences				32.67,57.56	
Control					
Gaila	111	98	-13		
Rajihar	51	40	-11		
Charkalehan	248	241	-7		
Kazichor	124	98	-26		
Laukathi	145	132	-13		
Borobighai	61	72	11		
Mean and standard deviation of the differences				-9.83,4.92	

*The p values assess the probability that the changes in utilization between the pre-pilot phase and the pilot phase in the Intervention Area were greater than in the Control Area. The t test was used for this calculation, with equal variances assumed
