# Supplementary material for: Learnings From a Pilot Study to Strengthen Primary Health Care Services: The Community-Clinic-Centered Health Service Model in Barishal District, Bangladesh
Source: Glob Health Sci Pract. 2021 Mar 15;9(Suppl 1):S179–89. doi: 10.9745/GHSP-D-20-00466 (PMC7971368; doi:10.9745/GHSP-D-20-00466)
Supplement: 20-00466-Uddin-Supplement2.pdf [file 20-00466-Uddin-Supplement2.pdf]

Supplement to: Uddin ME, George J, Jahan S, Shams Z, Haque N, Perry HB. Learnings from a pilot study to strengthen primary health care services: the community-clinic-centered health service model in Barishal District, Bangladesh. *Glob Health Sci Pract*. 2021;9(Suppl 1). https://doi.org/10.9745/GHSP-D-20-00466

Supplement 2. Further details of the implementation process for the Community-Clinic-Centered Health Service (CCHS) Model components

The implementation of the Community-Clinic-Centered Health Service (CCHS) Model consisted of several below components which were implemented through some sub-activities as shown in Table 1.

Table 1. Key components of Community-Clinic-Centered Health Service Model

Key components	Sub-activities
**CHW harmonization**	Harmonize job description for CHWs<br>Team training for CHWs<br>Community micro-planning meeting
**Community engagement**	Reformation of CGs and CSGs<br>Orientation of CG and CSG members
**Local government support**	Activation of UP Standing Committees<br>Orientation for UP bodies<br>Learning sharing and advocacy
**Accountability mechanism**	Community score card

CHW harmonization

Harmonized job description for CHWs

The Project developed harmonized job descriptions for different CHW cadres present in CC catchment areas: Health Assistants (HAs), Family Welfare Assistants (FWAs), and Community Health Care Provider (CHCPs). The harmonized job description outlined the main general functions and tasks of all CHW cadres, and the specific tasks in which CHWs can coordinate each other. The Project engaged a consultant to work with the National Steering Committee (NSC) on community health to develop harmonized job descriptions for CHWs. The harmonization process aimed to enable different cadres of CHWs to organize their work in a complementary fashion and facilitate alignment of their tasks. The NSC endorsed the new job descriptions and authorized their piloting in the Project area for one year. The District Coordination Committee circulated the new job descriptions to all CHWs in the Project area. The Project clarified the tasks of new job descriptions with all CHWs through an orientation where CHWs supervisors were also present. During regular field visits the Project staff and CHW

supervisors followed up with the CHWs to assess how well they were adapting to the new directives. The Project staff reported that the CHWs were developing their monthly work plans in coordination with their co-workers, were sharing reports with each other, and resolving issues through their combined efforts. Thus, harmonization and coordination among CHWs were strengthened in the Project area.

Team training

The Project organized team training for CHWs in collaboration with Save the Children's MaMoni Health System Strengthening Project and the National Institute of Population Research and Training (NIPORT), the designated government institute for building capacity of CHWs. Aiming at facilitation of harmonization among all CHW cadres (CHCPs, HAs, and FWAs along with any NGO CHWs working in the CC catchment area), the Project organized this team training. The key feature of the training course was- that all the CHWs working at in the CC catchment area received the same training together for the first time. Usually, since different CHWs cadres are administered by different authorities, capacity-building trainings are held separately. But, this time, they received the team training together in a common session that enabled them to share their views and learn together. The training duration was five days as a training center. The module covered the key contents of team building and harmonization among CHWs, joint planning, interpersonal communication skills, community participation and local government engagement.

The training was arranged for all CCs (187 CCs) of the DLL including the 25 CCs in the Project area. A total 609 CHWs (CHCPs, HAs, and FWAs) received this training. The Project worked with other collaborators, as mentioned above (MaMoni Project and NIPORT), developed the curriculum, arranged the Training of Trainers (TOT) workshop, and organized the training for CHWs. The facilitators were government and NGO health professionals – managers and supervisors of CHWs. Each training session consisted of the following sequence: lecture, discussion, brain storming, group discussion, question answer, case study, and video education materials. An evaluation of the training course reported that 85% participants thought the training would be helpful to them perform their job and duties at the field. Table 2 shows the characteristics of the participants in the team training.

Table 2. Characteristics of participants in the training course

Type of CHW	Sex		Total
	Male	Female	
Community Health Care Provider (CHCP)	75	96	171
Health Assistant (HA)	117	100	217
Family Welfare Assistant (FWA)	0	221	221

Total			609

The Project introduced monthly community micro-planning meetings (cMPM) at each CC in the Intervention Area. These were held following the session on immunizations. CHWs and their supervisors, one local government representative, along with one or two members from the CG and each of the CSGs would attend. Usually 12-15 persons participated in the meeting which was facilitated by a supervisor of CHWs. At that time, information was shared about newlyweds, newly pregnant mothers, maternal and child deaths, pregnant women with pregnancy-related complications, and women in need of contraceptives. Together they prepared a plan to resolve the identified issues. The meeting helped CHWs prepare their individual reports, ensuring uniformity and avoiding the duplication of data that they inserted into the national health information system (DHIS2). These cMPMs also enhanced harmonization and collaboration among CHWs and with the community.

Community engagement

The Project activated CC's Community Groups (CGs) and Community Support Groups (CSGs) across the district to strengthen community engagement of CC. The Project staff had observations and analysis that the CG-CSGs were not functioning as per guideline of MOHFW. There were irregularities in the frequency of monthly meetings, in the number of members attending, and in the adequacy of representativeness of members for the entire CC catchment area. With this observation, the Project used a social mapping tool that outline the whole catchment area and put dots as per the listed CG-CSG members according to their existence or household in the catchment area. This exercise revealed that most of the CG-CSG members live very close to the CC building. On an average, 40% of CC area had representation with CG or CSG members, and the rest did not have, as shown in the Map 1. The Project shared this at the respective CC and District Coordinating Committee meetings. Having been instructed by the District Coordinating Committee, the CCs reformed the CG-CSG members ensuring representation of all (100%) CC catchment area, as shown in Figure 1(map-2) and membership criteria, especially one third of women members in the group as per guideline. This reformation included new and active members in CG-CSGs. The Project oriented the CG-CSGs members on their functions and responsibilities in groups and toward CC operations.

Following the reformation and orientation, CG and CSGs members were attending meetings regular basis (CG meeting once in a month and CSGs met bi-monthly) as per government guideline. The CGs started oversight of clinic activities and liaised with their CSGs and other community members. CSGs held meetings and disseminated information to households about CC services. The Project also helped CHWs prepare a yearly calendar of health education sessions on maternal and child health, family planning and nutrition issues that the group members could disseminate to their surrounding community and households.

Supplement to: Uddin ME, George J, Jahan S, Shams Z, Haque N, Perry HB. Learnings from a pilot study to strengthen primary health care services: the community-clinic-centered health service model in Barishal District, Bangladesh. *Glob Health Sci Pract*. 2021;9(Suppl 1). https://doi.org/10.9745/GHSP-D-20-00466

The Project worked to establish an effective referral system from the CC to higher-level health facilities to improve access as well as to increase patient satisfaction. The Project trained CHWs on how to track the referred patients and worked with upazila health complex (UHC) to provide prompt service and to build up the trust of communities in the referral system. These interventions brought the following outcomes:

- CG and CSG members started generating funds and resources for the CC,
- They promoted information CC services in the community
- They arranged local transportation for referring poor patients from the CC to a higher-level facility

Supplement 2. Box 1. Responsibilities of the Community Group and the Community Support Group

Community Group

- Day-to-day operation of the CC
- Coordination with other providers and relevant stakeholders
- Ensuring security and cleanliness of the CC
- Monitoring and evaluation of CC performance
- Local-fund generation and transparent use of these funds
- Monitoring and evaluation of community participation

Community Support Group

- Make the community aware of services at CC
- Help poor, marginalized and vulnerable groups in getting services from the CC
- Help the community get emergency services
- Help the poor with referrals
- Disseminate health, nutrition and family planning messages in the community
- Help the Community Group with local fund generation
- Keep close contact with the CG

Local government support in community health

The Union Parishad (UP) is the lowest tier of government in rural Bangladesh. Each UP is made up of nine wards. Usually one village is designated as a ward. Each UP is supposed to have 13 Standing Committees on different issues including education, health, family planning, social welfare, disaster management, agriculture, fisheries, livestock, and rural communications. Each standing committee is comprised of 5-7 members headed by one elected representative. These committees are meant to prepare participatory planning, ensure implementation, monitoring, good governance, transparency, and accountability in rural areas.[1]

Supplement to: Uddin ME, George J, Jahan S, Shams Z, Haque N, Perry HB. Learnings from a pilot study to strengthen primary health care services: the community-clinic-centered health service model in Barishal District, Bangladesh. *Glob Health Sci Pract*. 2021;9(Suppl 1). https://doi.org/10.9745/GHSP-D-20-00466

The Project organized an orientation session for each UP's Education, Health and Family Planning Standing Committee, which is, as said comprised of 5-7 members from the community including one elected UP representative of the ward where the CC is situated. Through the orientation, the members learned about their responsibilities for community health programs and the ways they could support the CC, maintain liaison with community through CGs and CSGs, and mobilize resources from UP. The Project also oriented all of the local UP entities including the UP Chairman and all elected members of UP on their responsibilities for community health, and their obligation to allocate up to 15% of their annual budget for community health work as mandated by the government. Usually this allocation had been diverted to road repairs. To ensure local government support for community health, the Project worked in following areas in each union:

- Helped the Standing Committees related to health become more active
- Motivated the UP Chairman to form a Standing Committee for health if it did not exist
- Organized orientations for the Standing Committees and other UP entities

The Project arranged a knowledge-sharing session for all the UP entities as well as the district and upazila managers for health and family planning programs. The Project collaborated on this with Save the Children's other project, the MaMoni Health Systems Strengthening Project. During the learning and sharing meeting and workshop, the UP representatives expressed their motivation and inspiration for giving importance to and working on community health issues. Many representatives instantly committed to work for improving community health care services in their respective UP. The workshops inspired the UP entities to give more priority on maternal, neonatal and child health care through CCs. After returning back from the workshop, the UP entities held meetings with health and family planning staff, NGO staff and other stakeholders to begin to plan how they could improve the quality of services at the CCs. Most of the UPs started allocating funds from their annual budget for improving community health care.

The Project activated the latent UP's Education Health and FP Standing Committees in the Intervention Area. With support the local government across most of the 25 CCs in the Intervention Area, improvements were made in repairing of access roads to CCs; building boundary walls; installing tube wells; establishing electricity connections, lighting, furniture, roofing, and equipment; and making repairs. Solar panels were also installed in some CCs, and many CCs were provided with cleaners and security guards. Table 3 highlights the budget allocation for MNCHPFN issues in CCs by unions during the pilot interventions, (information gathered through project information system).

Table 3. Examples of Union Parishad budget allocations for maternal and child health and family planning activities for one year (October 2018-September 2019)

**Upazila**	**Union parishad**	**Amount (in USD)**	**Types of support**
**Babuganj**	Rahamatpur	$ 440	Connection road to CC, tube well repairs

Supplement to: Uddin ME, George J, Jahan S, Shams Z, Haque N, Perry HB. Learnings from a pilot study to strengthen primary health care services: the community-clinic-centered health service model in Barishal District, Bangladesh. *Glob Health Sci Pract*. 2021;9(Suppl 1). https://doi.org/10.9745/GHSP-D-20-00466

**Bakerganj**	Rangashree	$ 1,785	Solar power, water filter, chair, cleaning
**Gaurnadi**	Mahilara	$ 2,380	Table, earth filling, paint, connecting road, tube well, repairs, cleaning, electricity
**Banaripara**	Soliabagpur	$ 952	Repairs, safety precautions
**Wazirpur**	Bamrail	$ 595	Garden, earth filling, floor repairs with tiles
**Sadar**	Raypashakorapur	$ 137	Cleaning, repairs, installation of gate and boundary wall, tube well, chair

Spotlight on Mahilara Union: Leadership makes the difference

Being inspired by the Learning Sharing Workshop, Mr. Suikat Goha Piklu, the UP Chairman of Mahilara Union Parishad, Gournadi Upazila, Barishal, became an outspoken advocate for CCs. He supported the building of a road to the CCs, the planting of trees around the CC, repairing the patient waiting room, repainting the CC building and walls, and fencing around the CC grounds. He conducted weekly onsite monitoring of the CCs and CHWs and began to regularly update their work through social media. He created a Facebook page for all CCs in his union. This served as an information sharing platform regarding CC services and activities. In 2019, the Mahilara Union Parishad published a booklet on budget allocation reflecting the increased funding dedicated to CC improvements over the course of the Project. Mr. Suikat made health his first priority. As a result of his leadership and support, all CCs in his UP had a fresh look and an improved quality of services for the people in his UP.

Accountability mechanism: Introduction of Community Score Cards

The Project team introduced a Community Score Card (CSC) tool in the Intervention Area to provide feedback from the community regarding the quality of community-based care. The CSC approach consists of five phases (Appendix 2. Figure 2), which repeated themselves every six months during the Project implementation period.

Supplement 2. Figure 2. The introduction of Community Score Card

Supplement to: Uddin ME, George J, Jahan S, Shams Z, Haque N, Perry HB. Learnings from a pilot study to strengthen primary health care services: the community-clinic-centered health service model in Barishal District, Bangladesh. *Glob Health Sci Pract*. 2021;9(Suppl 1). https://doi.org/10.9745/GHSP-D-20-00466

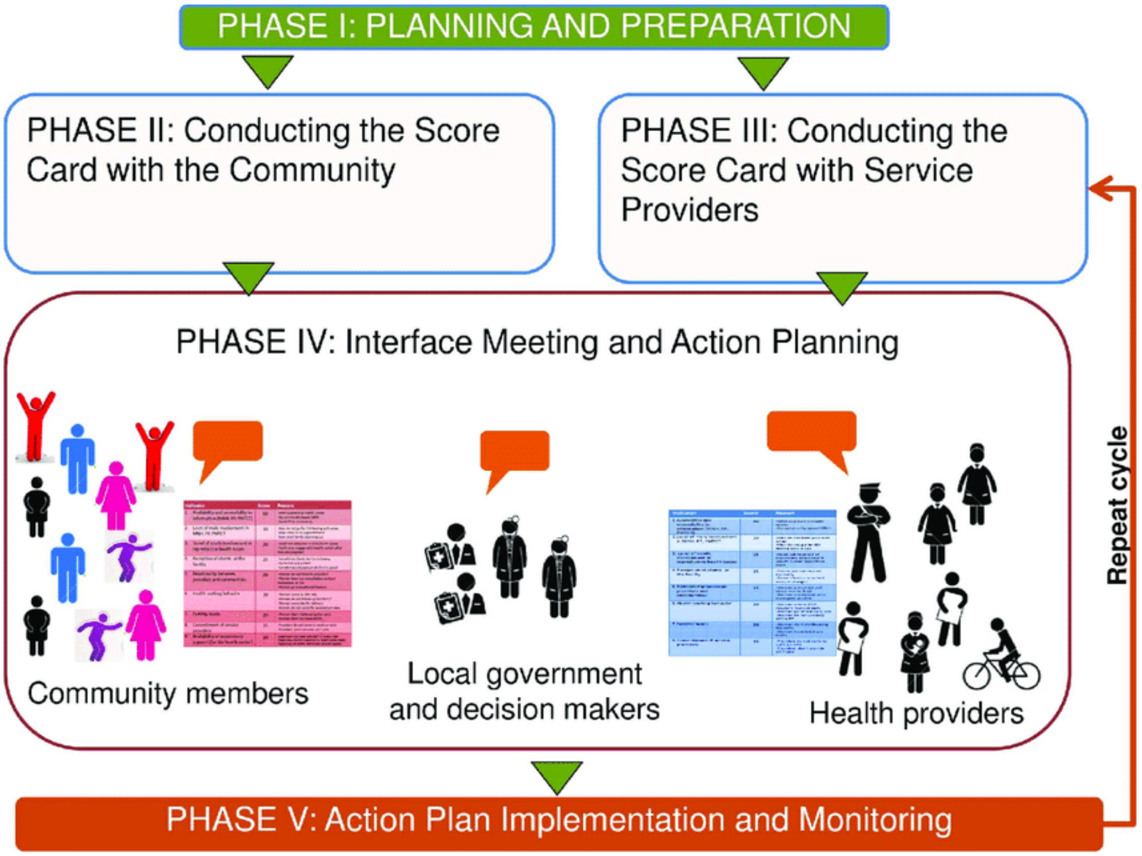

The Community Scorecard: Introduced in 13 CCs

The Project set the following key principles while facilitating the Community Score Card process.

- **Community centeredness** - engage the community through CGs and CSGs early and often in the process.
- **Build on the existing system -** facilitate the process using existing platforms in the community and health system.
- **User friendliness -** the CSC process facilitation should require minimum technical support and be easy to use.
- **Partnership with the local government and community -** engage the members of the local government to build a partnership for long-term sustainability.

During the implementation of the CSC process, the Project customized sub-activities for each phase. The CSC process involved community members, CHWs, local government representatives, and other health and local government officials. Implementation process shown in Appendix 2. Figure 3.

Supplement to: Uddin ME, George J, Jahan S, Shams Z, Haque N, Perry HB. Learnings from a pilot study to strengthen primary health care services: the community-clinic-centered health service model in Barishal District, Bangladesh. *Glob Health Sci Pract*. 2021;9(Suppl 1). https://doi.org/10.9745/GHSP-D-20-00466

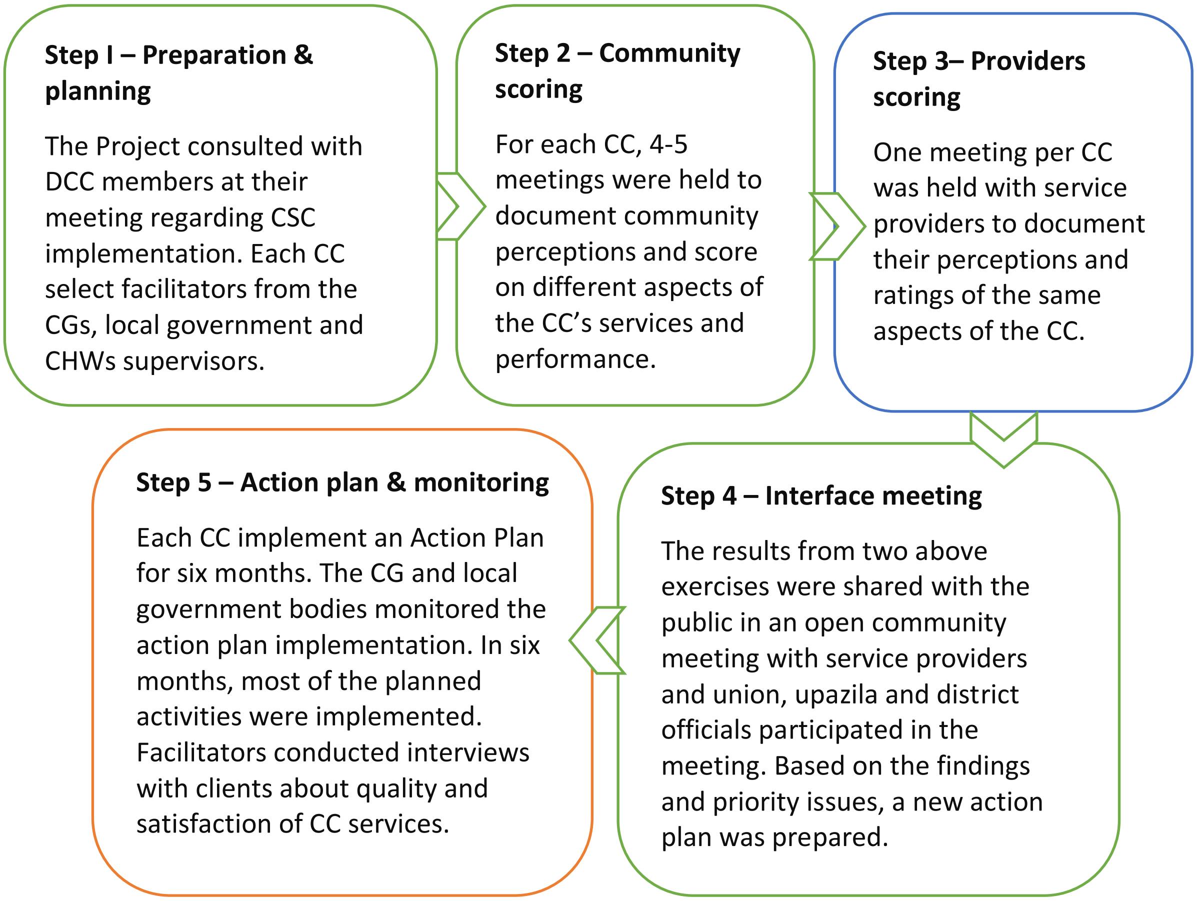

Supplement 2. Figure 3: CSC steps and activities carried out in CCHS Model

Key findings from CSC implementation

- Facility cleanliness and provider behaviors/ and skills were noted as key indicators of quality for communities
- Action plans yielded resource commitments from local government
- Scorecard process triggered more involvement from local governments in CC oversight

Lessons:

- Reformation of CG-CSGs enhanced community engagement and helped facilitate CSC process
- CSC tool seemed user friendly to the facilitators- accepted by community, stakeholders and service providers
- Accountability matters in two folds-community (demand side) and Service provider (supply side)

- Service providers, community and stakeholders realized their individual roles and level/provisions of accountability through CSC process

Recommendations

- Cost and operationalize the National Strategy for Community Health Workers, in particular strategic action 6.6 on effective community engagement for improving CHW performance and utilization, which covers engagement of relevant stakeholders, coordination, monitoring and accountability, and demand generation
- Establish platforms at community to national levels in which UP members and community systematically describe their process of activation and engagement with community health improvement, and the results it has achieved in their constituencies; disseminate to other UP members throughout the country through UP training platforms
- Develop a training package on Community Score Card implementation to enable other institutions and organizations to implement it without facilitation by outsider organization

Organize and facilitate digitally accessible resource groups of experienced local government representatives, CHWs, and CG/CSG members who can provide remote informal guidance to, and answer questions from, their counterparts.

REFERENCE

1. Islam T. *Understanding the Effectiveness of Union Parishad Standing Committee: A Perspective on Bangladesh*. 2017. https://core.ac.uk/reader/161929630
