# Supplementary material for: Learnings From a Pilot Study to Strengthen Primary Health Care Services: The Community-Clinic-Centered Health Service Model in Barishal District, Bangladesh
Source: Glob Health Sci Pract. 2021 Mar 15;9(Suppl 1):S179–89. doi: 10.9745/GHSP-D-20-00466 (PMC7971368; doi:10.9745/GHSP-D-20-00466)
Supplement: 20-00466-Uddin-Supplement1.pdf [file 20-00466-Uddin-Supplement1.pdf]

Supplement to: Uddin ME, George J, Jahan S, Shams Z, Haque N, Perry HB. Learnings from a pilot study to strengthen primary health care services: the community-clinic-centered health service model in Barishal District, Bangladesh. *Glob Health Sci Pract*. 2021;9(Suppl 1). https://doi.org/10.9745/GHSP-D-20-00466

Supplement 1. An overview of Bangladesh's community health system

Bangladesh has an extensive three-tiered network of government facilities at tertiary, secondary, and primary levels. However, utilization of government district hospitals and health centers is low. There is a strong mix of public, private, nongovernmental organization, and traditional providers, leading to a pluralistic health system that is also minimally regulated.[1,2] The public and private sectors have a porous boundary: many government doctors also work part-time in private facilities or in their own private office, often bringing patients from a government facility who can pay to a private facility where these same doctors can provide better care for a fee. Village doctors (informally trained providers who practice allopathic medicine) are the dominant providers of ambulatory curative care in the community.[3]

Bangladesh embarked on a major health sector reform in 1998 aiming to increase efficiency, sustainability, and integration of the existing health system and services.[4] In 1998, Bangladesh launched the Community-Based Health Care Program that introduced the community clinic (CC) concept "to extend quality primary health care services to rural people all over the country.''[5] The CC was envisioned as a hub for community health workers (CHWs) to provide services to the community. Family welfare assistants (FWAs), long-time CHWs employed by the Directorate General of Family Planning, and health assistants (HAs), long-time CHWs employed by the Directorate General of Health Services, had focused on delivering family planning and immunization services, respectively, at the household level. With the establishment of the CCs, the FWAs and the HAs were expected to spend half of their time at the CC.

Each CC is staffed by a community health care provider (CHCP), also considered to be a CHW. The FWAs and the HAs, while working in the CC, would support the CHCP. NGO-recruited CHWs would also work in the CC in a similar fashion. NGO CHWs are usually located in areas where FWAs and HAs are not available and also in very hard-to-reach areas. By 2018, the government had constructed 13,779 CCs and staffed them with 13,507 CHCPs, 19,583 FWAs, and 15,420 HAs.[6,7]

Community-level service delivery in Bangladesh is managed and coordinated across the national, district, upazila, union, and ward levels. Each administrative level has a distinct role in supporting community policy and program efforts. At the national level, the MOHFW is responsible for the implementation, management, coordination and regulation of health-related policies, programs, and activities. Its core functions are policy and strategy planning, monitoring, and management of budgets, information, reform, and funding. The Directorate General of Health Services and the Directorate General of Family Planning are the agencies within the MOHFW that provide health services in rural areas. The Ministry of Local Government, Rural Development and Co-operatives works with the MOHFW to implement health services in urban areas. National bodies also manage the appointment, transfer, posting, and salary of community health providers. At the ward level, community groups (CGs) are responsible for running CCs, including their maintenance, security, fundraising, and community mobilization. Community Support Groups support the community groups and ensure that the level of services provided is acceptable.[8]

Supplement to: Uddin ME, George J, Jahan S, Shams Z, Haque N, Perry HB. Learnings from a pilot study to strengthen primary health care services: the community-clinic-centered health service model in Barishal District, Bangladesh. *Glob Health Sci Pract*. 2021;9(Suppl 1). https://doi.org/10.9745/GHSP-D-20-00466

Figure 1 summarizes Bangladesh's government health structure, including service delivery points, key actors, and managing bodies at each level.

Figure 1. Bangladesh's health system structure

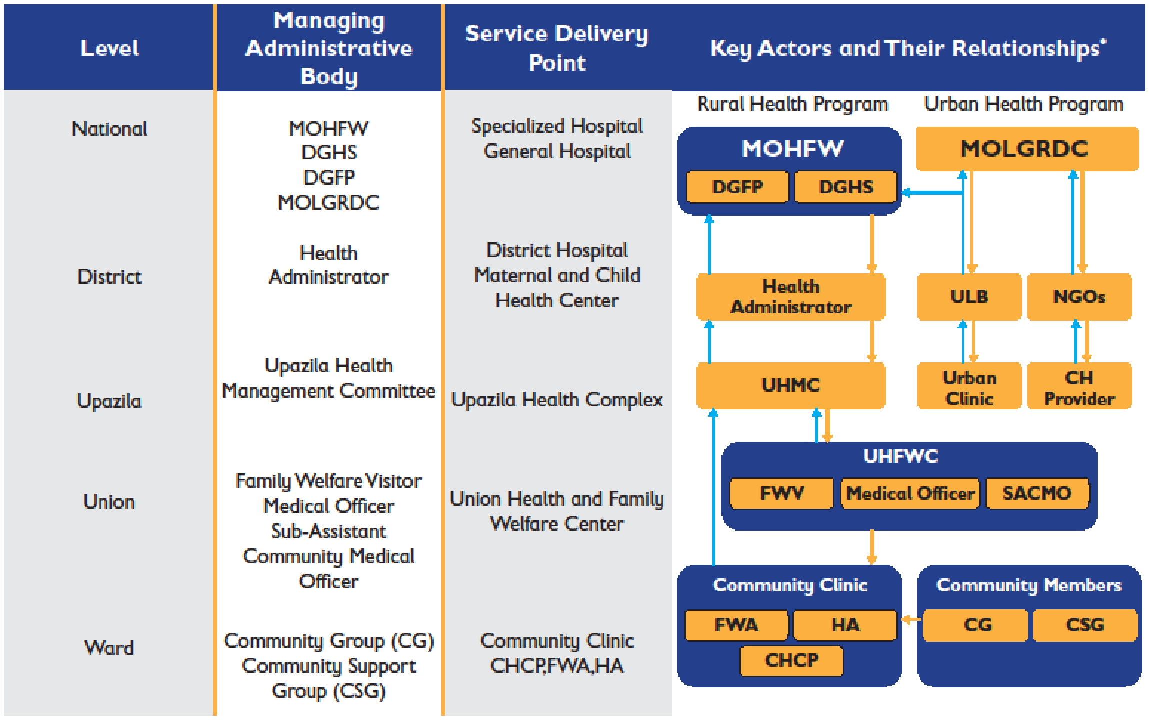

*NGO provide support at all levels and work in close collaboration with the government in community health planning and implementation

Glossary: FWA-Family Welfare Assistant; HA- Heatlh Assistant; CHCP-Community Health Care Provider; CH-Community Health; MOHFW-Ministry of Health and Family Welfare; DGHS-Directorate General of Health Services; DGFP-Directorate General of Family Planning; MOLGRDC-Ministry of Local Government Rural Development and Cooperatives; ULB-Urban Local Body

Rural Health Program →
Flow of community level data →

The CC is a public-private partnership. All the CCs have been constructed on community-donated land while construction, medicines, service providers, logistics, and all other inputs are provided by the Government of Bangladesh. CCs are managed both by the community and the government through community groups. CCs provide a wide range of services, including maternal and neonatal health care services, Integrated Management of Childhood Illness, reproductive health and family planning services, immunizations, nutritional education and micronutrient supplementation, health education and counseling, screening for chronic non-communicable diseases, treatment of minor ailments and common diseases, first aid, and referral of patients to higher facilities.

CCs face many challenges such as shortages of staff, supplies and equipment as well as weaknesses in community engagement and accountability. Community-based health services lack coordination, hindering effective program implementatio.[9,10] Additionally, insufficient continuous support from the local government and the community acted as barriers to achieving a desired level of coverage and quality of health services.[5]

Supplement to: Uddin ME, George J, Jahan S, Shams Z, Haque N, Perry HB. Learnings from a pilot study to strengthen primary health care services: the community-clinic-centered health service model in Barishal District, Bangladesh. *Glob Health Sci Pract*. 2021;9(Suppl 1). https://doi.org/10.9745/GHSP-D-20-00466

REFERENCES

1. Joardar T, Javadi D, Gergen J, Perry H. The Government Family Welfare Assistants, and Community Health Care Providers in Bangladesh. In: Perry H, ed. National Community Health Programs: Descriptions from Afghanistan to Zimbabwe. Washington. DC: USAID/Jhpiego/Maternal and Child Survival Program; 2020.

2. Joardar T, Javadi D, Gergen J, Perry H. The BRAC *Shasthya Shebika* and *Shasthya Kormi* Community Health Workers in Bangladesh. In: Perry H, ed. Health for the People: National Community Health Programs from Afghanistan to Zimbabwe. Washington. DC: USAID/Jhpiego/Maternal and Child Survival Program; 2020.

3. Mahmood SS, Iqbal M, Hanifi SMA, Wahed T, Bhuiya A. Are 'Village Doctors' in Bangladesh a curse or a blessing? BMC Int Health Hum Rights. 2010;10(1):18. doi:10.1186/1472-698X-10-18. Medline

4. Ahsan KZ, Streatfield PK, Ijdi RE, Escudero GM, Khan AW, Reza MM. Fifteen years of sector-wide approach (SWAp) in Bangladesh health sector: an assessment of progress. Health Policy Plan. 2016;31(5):612-623. doi:10.1093/heapol/czv108. Medline

5. Government of the People's Republic of Bangladesh, Ministry of Health and Family Welfare (MOHFW). *Independent Evaluation of Community Based Health Services in Bangladesh*. MOHFW, Directorate General of Health Services, Community Based Health Care programme, World Health Organization; 2019. Accessed January 20, 2021. https://www.who.int/docs/default-source/searo/bangladesh/pdf-reports/2019-20/cbhs---report-2019.pdf

6. Government of Bangladesh. Bangladesh National Strategy for Community Health Workers (2019-2030). 2019. https://www.healthynewbornnetwork.org/hnn-content/uploads/Bangladesh__Community-Health-Workers.pdf (accessed 7 December 2020.

7. MOHFW/Bangladesh. Health Bulletin 2018. 2018. https://dghs.gov.bd/images/docs/Publicaations/HB%202018%20final.pdf (accessed 7 December 2020.

8. Advancing Partner and Communities. Community Health System Catalog Country Profile: Bangladesh. 2017. https://www.exemplars.health/-/media/resources/community-health-workers/bangladesh/community-health-systems-catalog-country-profile-bangladesh.pdf.

9. Bangladesh Planning Commission. Bangladesh Seventh Five Year Plan, FY2016-2020. 2015. http://nbr.gov.bd/uploads/publications/154.pdf (accessed 7 December 2020.

10. MOHFW/Bangladesh. Community Based Health Care. http://www.communityclinic.gov.bd/ (accessed 7 December 2020.
